# Expression profiling of *Chrysanthemum crassum* under salinity stress and the initiation of morphological changes

**DOI:** 10.1371/journal.pone.0175972

**Published:** 2017-04-24

**Authors:** Zhiyong Guan, Yitong Feng, Aiping Song, Xiaomeng Shi, Yachao Mao, Sumei Chen, Jiafu Jiang, Lian Ding, Fadi Chen

**Affiliations:** College of Horticulture, Institution of Nanjing Agricultural University, City of Nanjing, State of Jiangsu Province, Country of China; Chinese Academy of Sciences, CHINA

## Abstract

*Chrysanthemum crassum* is a decaploid species of *Chrysanthemum* with high stress tolerance that allows survival under salinity stress while maintaining a relatively ideal growth rate. We previously recorded morphological changes after salt treatment, such as the expansion of leaf cells. To explore the underlying salinity tolerance mechanisms, we used an Illumina platform and obtained three sequencing libraries from samples collected after 0 h, 12 h and 24 h of salt treatment. Following *de novo* assembly, 154,944 transcripts were generated, and 97,833 (63.14%) transcripts were annotated, including 55 Gene Ontology (GO) terms and 128 Kyoto Encyclopedia of Genes and Genomes (KEGG) pathways. The expression profile of *C*. *crassum* was globally altered after salt treatment. We selected functional genes and pathways that may contribute to salinity tolerance and identified some factors involved in the salinity tolerance strategies of *C*. *crassum*, such as signal transduction, transcription factors and plant hormone regulation, enhancement of energy metabolism, functional proteins and osmolyte synthesis, reactive oxygen species (ROS) scavenging, photosystem protection and recovery, and cell wall protein modifications. Forty-six genes were selected for quantitative real-time polymerase chain reaction detection, and their expression patterns were shown to be consistent with the changes in their transcript abundance determined by RNA sequencing.

## Introduction

Salt can diffuse from underground soil, wind and rain can carry salt from the ocean, and human overuse of chemical fertilizers also causes salty soils [1, 2]. These factors have increased the amount of land affected by salt. According to a study by the Food and Agriculture Organization (FAO) in 2011, more than 800 million hectares of land were estimated to be affected by salt around the world.

Salinity can influence the survival and growth of plants. Salinity weakens photosynthesis and enhances the respiration of plants, thereby slowing their growth rate [3]. Moreover, salinity can cause osmotic stress, which disturbs water assimilation by plants [4] and can lead to the accumulation of ROS, which, in excessive levels, damage plant cell membranes [5]. Salinity can also cause Na^+^ to accumulate, which disrupts the assimilation of other cations, such as K^+^, and disturbs normal plant cell functions [6]. Under salinity stress, plants regulate the expression levels of a series of genes involved in stress adaptation. Some of these genes, including those involved in plant hormone regulation (such as *PP2C* and *MYC2*) [7], osmolyte synthesis (*TP* and *P5CS*) [8], ROS scavenging (*SOD* and *CAT*) [9] and salt exclusion and compartmentalization (*HKT*, *SOS1* and *NHX*) [6], have been widely studied in response to salinity stress. The tissues of some plants also display morphological changes under salinity stress. For example, the leaves of *Populus euphratica* become greatly incrassated under salinity stress [10], and some cell wall modification proteins, such as *HRGP* and *XET*, are considered to be responsible for these changes [11].

We previously found that most species of the *Chrysanthemum* genus planted in saline soil display unhealthy phenotypes, such as reduction of the growth rate, tissue wilt, leaf necrosis and abscission, and interference with flowering [12]. Such negative impacts of salinity have an adverse influence on the ornamental quality of chrysanthemums and disturb breeding by researchers. However, the salinity tolerance of chrysanthemums varies among different species, and *C*. *crassum* is an elite germplasm that can survive and grow well under moderate salt stress (120 mM NaCl) conditions [13]. In this study, we found that under long-term moderate salt stress, *C*. *crassum* could survive and maintain an ideal growth rate; interesting morphological changes also occurred, such as the cells of salt-treated leaves expanded, and the leaves were elongated and incrassated compared with the controls. Additionally, the accumulation of Na^+^ in the functional leaves of *C*. *crassum* increased slowly and eventually stopped increasing over time. All of changes would be initiated and controlled by genes that respond to salinity stress at an early stage. Therefore, illuminating the mechanisms involved in the response of *C*. *crassum* to salinity stress may help to identify the genes that contribute to salinity tolerance in plants, and it is important to determine how salt initiates morphological changes.

Next-generation sequencing (NGS), with its excellent characteristics of cost-effectiveness and high throughput [14], is the most promising method for studying expression profiles in non-model plants that lack whole-genome sequencing data [15]. In this study, we used NGS to examine the changes in transcriptional levels in *C*. *crassum* after salt treatment. We also aimed to determine the essential metabolic pathways and key genes that contribute to salinity tolerance.

Given the remarkable phenotypic changes in the leaves of *C*. *crassum*, we chose to sequence RNA from the leaves. According to previous studies [8], the salinity tolerance traits of leaves are mainly related to osmotic tolerance and tissue tolerance; the former is instantaneously triggered and related to sensing and signaling mechanisms, while the latter is related to the compartmentalization of salt to intercellular spaces and vacuoles and may be associated with processes involving ion transporters, proton pumps and synthesis of compatible solutes. In this study, we sampled the leaves of *C*. *crassum* at 0 h, 12 h and 24 h after salt treatment, and performed RNA-seq. As we determined the transcriptomes of these samples, we analyzed the genes and mechanisms that contributed to salinity tolerance, especially osmotic and tissue tolerance, and summarized the strategies adopted by *C*. *crassum* under salinity stress. In addition, responsive genes can be selected for breeding into other species of chrysanthemum to improve their salinity tolerance. Thus, this study presents transgenic experiments aiming to improve salinity tolerance.

## Materials and methods

### Plant materials and growth conditions

Plants were obtained from the Chrysanthemum Germplasm Resource Preservation Center of Nanjing Agricultural University, China. Shoot cuttings of *C*. *crassum* were rooted and grown in matrix (vermiculite: perlite: nutritive soil = 1:1:1). Rooted seedlings at the 6–7 leaf stage were selected and then transplanted into plastic containers (volume 20 L) filled with diluted (1:2) Hoagland nutrient solution, with aeration for 24 h•d^–1^. The plants were maintained in a greenhouse with a normal photoperiod and sunlight, an average temperature of 25°C and relative humidity of 70%; all solutions were renewed every two days. After one week, one set of plants grown in Hoagland solution alone was kept as a control (CK), and salt treatment (S) was performed by supplementing the nutrient solution with 120 mM NaCl.

### Recording of morphological changes after salt treatment and paraffin sectioning

At 0 d, plants that showed similar sizes and shapes were selected from the salt treatment and control groups and photographed. Photographs of these plants were subsequently taken at 5 d, 10 d, and 20 d. At the same time points, the fourth unfolded leaves of the plants were traced on paper without injuring them.

The fourth unfolded leaves numbered from the tops of both salt-treated and control plants were picked (at 0 d, 5 d, 10 d, and 20 d), cut into pieces of 0.3 * 0.3 cm, then immediately fixed in FAA solution. The samples were subsequently dehydrated through an alcohol series, infiltrated with xylene, and embedded in paraffin wax. Transverse sections were cut to a thickness of 25 μm and stained with 0.1% toluidine blue solution (w/v). The sections were finally observed under an Olympus BX41 microscope [16].

### Determination of Na^+^ and K^+^ levels

The unfolded leaves numbered from the top of both salt-treated and control plants were collected (approximately 3 g, at 0 d, 5 d, 10 d, 20 d, and 30 d). The samples were then oven-dried at 70°C for 48 h, after which 50 mg DW was digested in 35% HNO_3_. Na^+^ and K^+^ was resuspended in 10 ml of HCl (0.1 N) and the solutions were filtered. Ions were quantified via flame atomic absorption spectrophotometry (VARIAN spectra-300) [17]. Three replicates of each sample were examined.

### RNA extraction and quality determination

The prepared samples were collected at 0 h, 12 h, and 24 h, and the leaves were immediately harvested, snap-frozen in liquid nitrogen and stored at −80°C. A Total RNA Isolation System (TaKaRa Bio, Tokyo, Japan) was employed to extract RNA from the samples according to the manufacturer’s protocol. The quality of the RNA was verified using a 2100 Bioanalyzer RNA Nanochip (Agilent, Santa Clara, CA), and its concentration was determined using an ND-1000 Spectrophotometer (NanoDrop, Wilmington, DE). At least 20 mg of RNA from three replicates were pooled in an equimolar fashion [18].

### Illumina cDNA library preparation and sequencing

Sequencing based on an Illumina platform (San Diego, CA) was performed at the Beijing Genomics Institute (Shenzhen, China). Briefly, poly(A) mRNA was isolated using beads coated with oligo (dT). Fragmentation buffer was added to fragment the mRNA to a size of 100–400 bp. The fragments were used for the synthesis of first-strand cDNA employing random hexamer primers. Second-strand cDNA was synthesized with the SuperScript Double-Stranded cDNA Synthesis kit (Invitrogen, Camarillo, CA). The cDNA was then purified using a QiaQuick PCR Extraction Kit (Qiagen, Hilden, Germany) and resolved with EB buffer for end reparation and poly(A) addition. The products were ligated with each another using sequencing adapters, and after agarose gel electrophoresis, a suitable size range of fragments was selected for PCR amplification. The resulting library was sequenced using the Illumina HiSeqTM 2000 system [18].

### Data filtering and de novo assembly

The image data output from the sequencing system was transformed into raw reads and stored in FASTQ format. These data were filtered to remove raw reads that included adapter sequences, had more than 5% unknown nucleotides or that were of low quality (the percentage of reads with quality value ≥ 10 was more than 20%). Transcriptome *de novo* assembly was carried out with Trinity [19]. The resulting Trinity sequences were considered unigenes. After sequence splicing and redundancy removal, gene family clustering was performed, and the unigenes were divided into two classes. In one cluster (with the prefix “CL” followed by the cluster id), there were several unigenes showing similarity of greater than 70%. The others were singletons (which have the prefix “Unigene”). BLASTX [20] alignment between each unigene sequence and those registered in the Nr (non-redundant protein database, NCBI), Nt (non-redundant nucleotide database, NCBI), Swiss-Prot, and GO (gene ontology, http://www.geneontology.org/) databases was performed, and the best alignments for inferring the directionality of each unigene were determined. For the best alignments in which the outcome from the various databases conflicted with one another, the priority order applied was Nr, followed by Swiss-Prot. The software tool ESTScan [21] was used to assign directionality.

### Gene annotation and analysis

Functional annotation was assigned using the protein (Nr and Swiss-Prot) and GO databases. BLASTX was employed to identify related sequences in the protein databases based on an E-value of less than 10^−5^. The annotations acquired from Nr were processed by using the Blast2GO program [22] to obtain the relevant GO terms, which were then analyzed with WEGO software [23] to assign a GO functional classification and to illustrate the distribution of gene functions.

### Prediction of unigene coding regions

Unigenes were aligned to protein databases using BLASTX with the following order of priority: Nr, Swiss-Prot, and KEGG. The proteins with highest ranks in the BLAST results were selected to determine the coding region sequences of the unigenes, and the sequences of the coding regions were then translated into amino sequences using the standard codon table. Thus, both the nucleotide sequences (5' -> 3') and amino acid sequences of the Unigene coding region were acquired. Unigenes that could not be aligned to any database were scanned by ESTScan to determine the direction of the nucleotide sequence (5’ -> 3’) and the amino acid sequence of the predicted coding region [21].

### Gene validation and expression analysis

Samples were prepared, and total RNA was extracted using the method indicated above. A total of 16 unigenes that responded to salt stress were chosen for validation. Three independent biological replicates of each sample were used in the analysis. A set of gene-specific primer pairs was designed using Primer3 software [24]. Reverse transcription was performed with M-MLV reverse transcriptase (TaKaRa). The relative expression of these genes was determined through qRT-PCR analysis using a SYBR^®^ Green reaction kit (TaKaRa), with the elongation factor 1-alpha gene as a reference. Then, a Mastercycler ep realplex device (Eppendorf, Hamburg, Germany) was used to run the qPCR assays. The transcription data were calculated using the −ΔΔCt method [25].

## Results

### Morphological changes of *C*. *crassum* and Na^+^ accumulation in leaves under salinity stress

**Fig 1:**
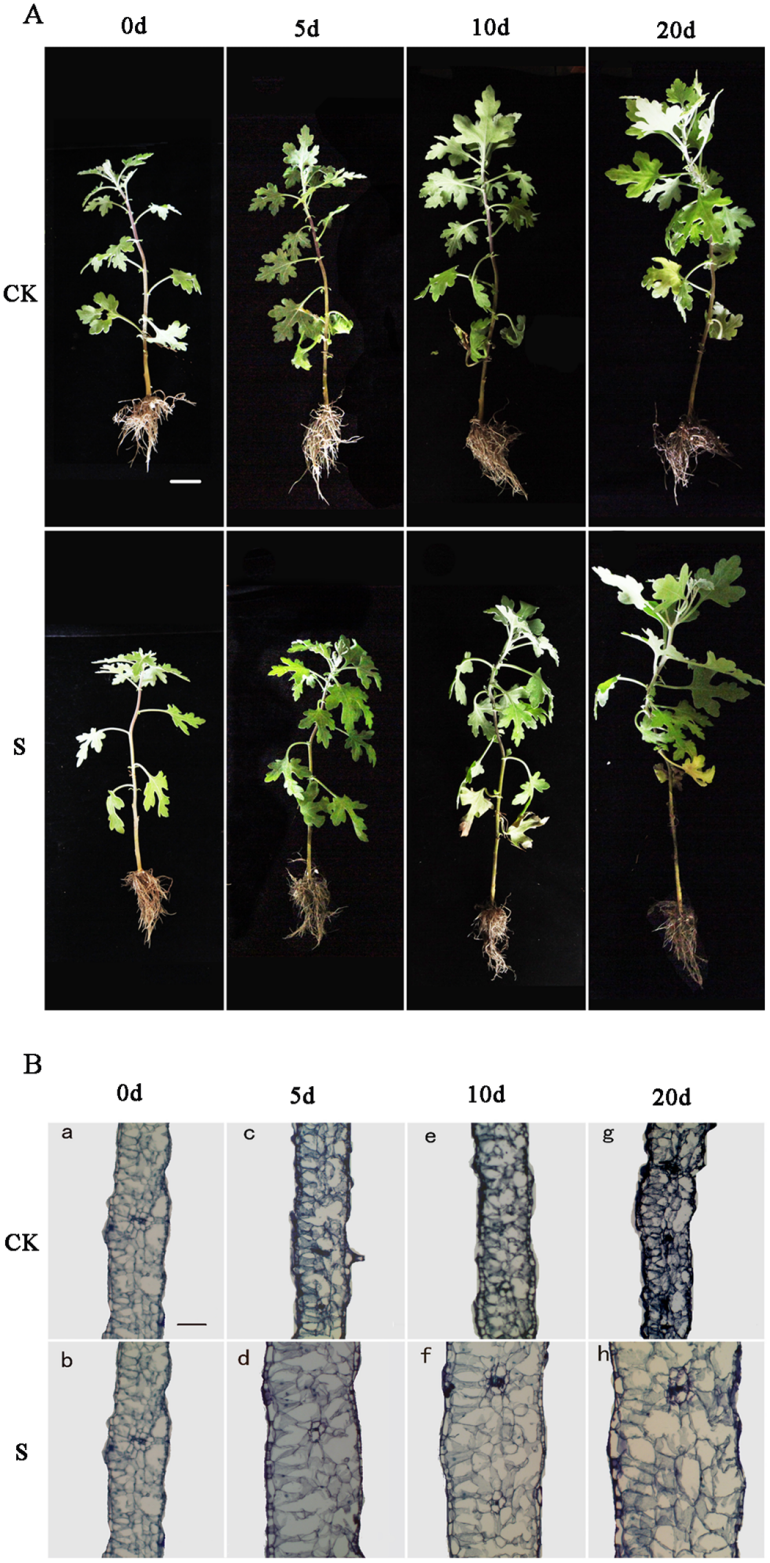
Growth conditions of *Chrysanthemum crassum* (A) and the incrassation of *C*. *crassum* leaves (B) under salinity stress. S = Salt, CK = Control; bar = 3 cm (A) and 100 μm (B).

*C*. *crassum* showed remarkable tolerance under salinity stress, as the salt-treated plants grew almost as fast as the controls (Fig 1A), with only the leaves near the roots wilting, revealing the damage due to salinity stress. As shown in Fig 1A, the leaves treated with salt grew differently from the controls; therefore, we chose salt-treated and control leaves of the same size and followed their growth by tracing the outlines of the leaves without injuring them at 0 d, 5 d, 10 d, and 20 d. The shapes of the leaves are shown in S1 Fig. It was apparent that the leaves that were treated with salt were larger than those of the controls, and the analysis of paraffinic slices of leaves (Fig 1B) showed that the leaves that were salt-treated were thicker than the leaves of the controls. This incrassation was mainly caused by cell expansion and not cell replication, as the number of cell layers was not increased. The morphological changes that occur in *C*. *crassum* under salinity stress have rarely been studied; however, they may be relevant to the remarkable salinity tolerance of *C*. *crassum*.

**Fig 2:**
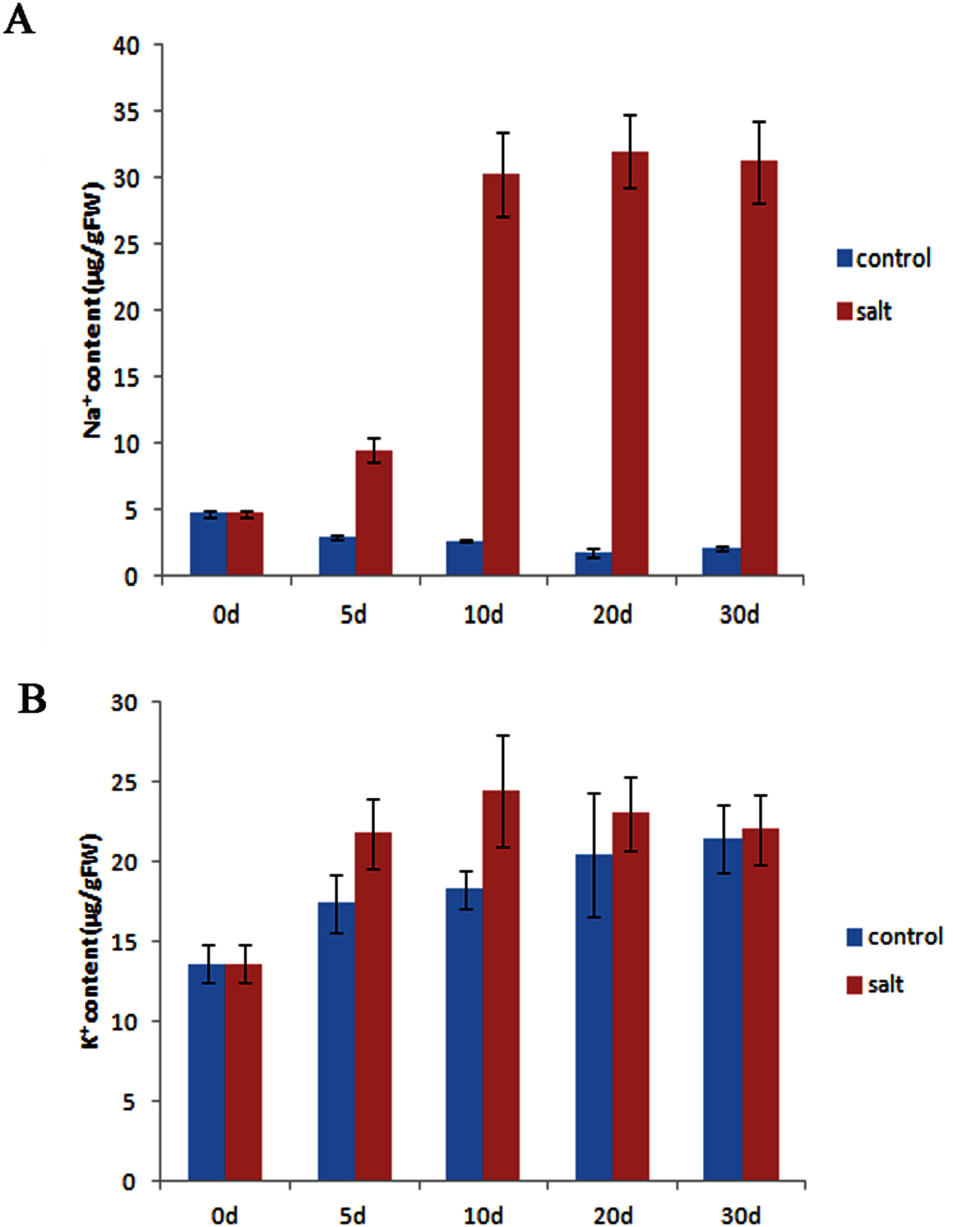
Changes in the Na^+^ (A) and K^+^ (B) contents of *Chrysanthemum crassum* leaves under salt stress. The Na^+^ and K^+^ contents were measured from the leaves collected at 0 d, 5 d, 10 d, 20 d, and 30 d after salt treatment. (n = 3, vertical bars indicate standard deviation [SD]).

The accumulation of Na^+^ in the leaves of salt-treated *C*. *crassum* was slow, requiring days to reach a peak, and a balance was maintained around the peak as time passed (Fig 2A). The K^+^ content of leaves also increased under salinity stress. These behaviors are beneficial to the survival and growth maintenance of *C*. *crassum* under salinity stress. The transcriptomes of the three samples helped us to determine the implications of these behaviors and identify which strategies *C*. *crassum* employs under salinity stress.

### *De novo* assembly and quantitative assessment of RNA sequences

To investigate the global gene expression profiles of the *C*. *crassum* transcriptomes at different stages of salinity stress, we constructed three cDNA libraries using leaves from hydroponic cutting seedlings treated with 120 mM NaCl for 0 h, 12 h, and 24 h.

Sequencing was carried out on an Illumina platform; a total of 29,831,115,240 nt were generated, and 346,363,148 raw reads were obtained. After removing low-quality regions, adapters, and contamination, we obtained a total of 331,456,836 clean reads with Q20 > 98.34% and a GC percentage between 43.38% and 43.75% (Table 1).

**Table 1:** Statistical summary of sequencing and assembly results.

Samples	0 h	12 h-S	24 h-S
Total Raw Reads	69,891,736	68,980,120	68,492,896
Total Clean Reads	67,121,284	65,942,222	65,383,846
Total Clean Nucleotides (nt)	6,040,915,560	5,934,799,980	5,884,546,140
Q20 percentage	98.40%	98.34%	98.35%
N percentage	0.00%	0.00%	0.00%
GC percentage	43.46%	43.39%	43.75%
Total Contigs	160,031	172,631	172,325
Total Length (nt)	52,499,663	54,800,646	54,903,879
Mean Length (nt)	328	317	319
N50	603	546	552
Total Unigenes	94,271	104,023	105,665
Total Length (nt)	64,918,123	71,004,381	73,626,732
Mean Length (nt)	689	683	697
N50	1254	1250	1248
All Unigenes	Total Number: 154,944; Total Length (nt): 167,656,676; Mean Length (nt): 1.082; N50: 1,661		

Note: The Q20 percentage is the proportion of nucleotides with a quality value > 20. The N percentage is the proportion of unknown nucleotides in clean reads. The GC percentage is the proportion of guanidine and cytosine nucleotides among total nucleotides. N50 is 50% of the assembled bases that were incorporated into sequences with a length of N50 or longer.

In the assembly results, 154,944 unigenes were detected; the total length of the unigenes was 167,656,676 nt; the average length was 1,082 nt; and the N50 was 1,661 nt. We generated 160,301, 164,718, and 174,748 contigs for the samples obtained at 0 h, 12 h, and 24 h after salt treatment, with average lengths of 328, 321, and 318 nt, respectively, and average N50 lengths of 603, 556, and 546 (Table 1).

### Sequence annotation and functional categories of annotated sequences

For the annotation of functional information in the assembled unigenes, we used several applications, including information on protein sequence similarities, GO and Kyoto Encyclopedia of Genes and Genomes (KEGG) pathways. All of the sequences were successively subjected to BLAST searches in the NCBI Nr database, the Nt database, and the Swiss-Prot protein database, with E-values < 1e-5. Using the best hits provided by BLAST, the most reasonable functions were assigned to the sequences. After functional annotation analysis, we obtained 93,296, 68,322, 65,127, 60,906, and 55,754 unigenes annotated to the NR, NT, Swiss-Prot, KEGG, and GO databases, respectively, totaling 97,833 annotated unigenes.

The E-value distribution of the top hits in the Nr database showed that 50.74% of the sequences were mapped to the known genes in plants with best hits (E-value < 1e-45, mean identity = 53.21%; S2A Fig), and approximately 15.88% of the unigenes hit deposited sequences with a similarity > 80% (S2B Fig). Approximately 72.32% of the annotated unigenes could be assigned with a best score to deposited sequences from the top seven species: *Vitis vinifera* (24.82%), *Lycopersicon esculentum* (15.95%), *Amygdalus persica* (8.06%), *Ricinus communis* (7.24%), *Populus balsamifera subsp*. *trichocarpa* (7.06%), *Fragaria vesca subsp*. *vesca* (4.99%), and *Glycine max* (4.22%; S2C Fig).

**Fig 3:**
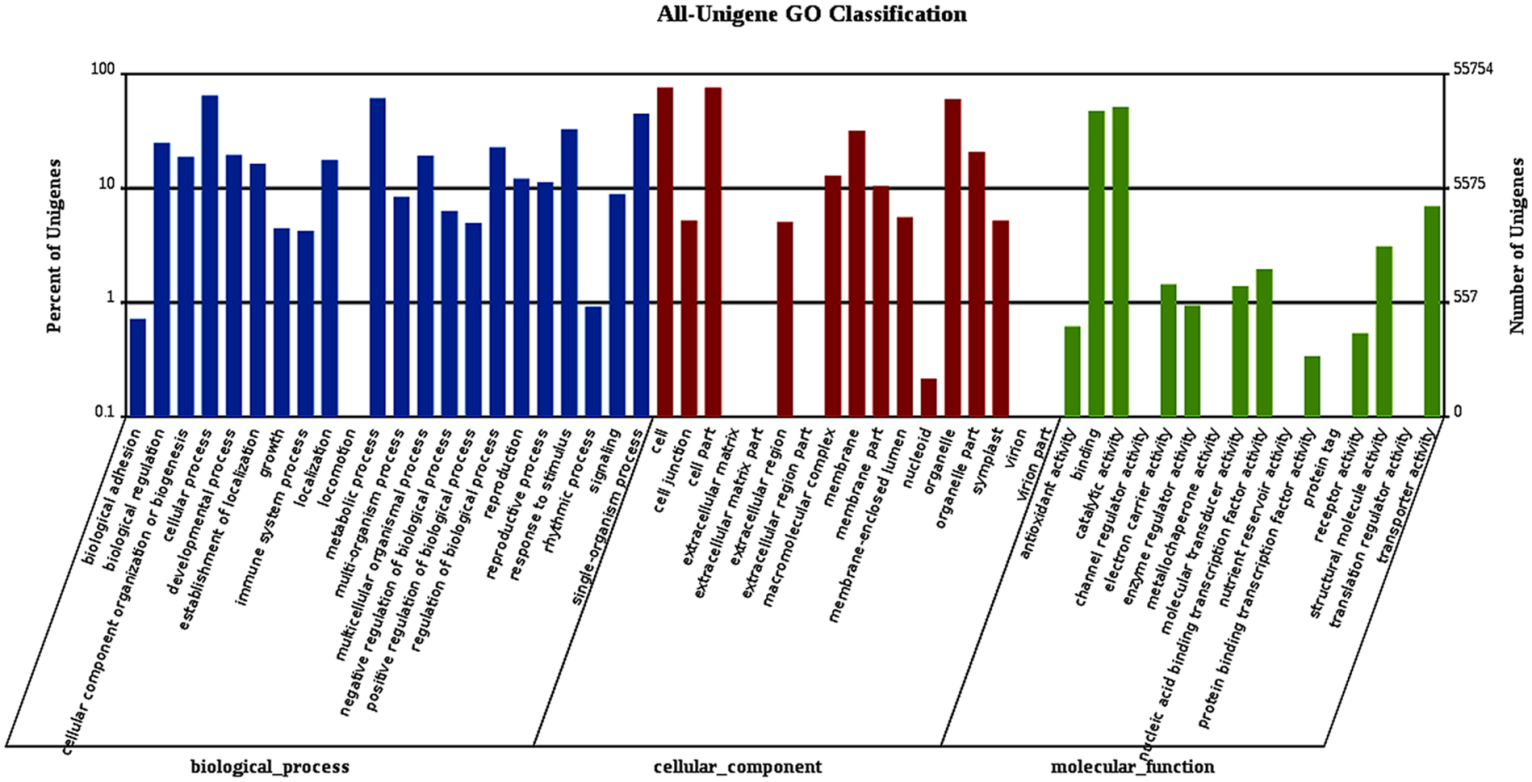
Histogram presentation of all unigene Gene Ontology classifications. The results are summarized in three main categories (biological processes, cellular components, and molecular functions) and 55 subcategories. The left Y-axis represents the percentage of a specific category of genes in each main category, and the right Y-axis represents the number of genes in a category.

Regarding the functional categories of the 154,944 unigenes, a total of 55,754 unigenes were assigned at least one GO term, which are mainly divided into three categories: biological processes, molecular functions, and cellular components. Among the 22 GO terms corresponding to biological processes, the terms, “cellular processes”, “metabolic processes”, “single-organisms”, “response to stimulus”, and “biological regulation” were significantly overrepresented, while “cell”, “cell part”, and “organelle” were significantly overrepresented among the 17 GO groups of cellular components, and “catalytic activity” and “binding” were significantly overrepresented among the 16 GO groups of molecular functions (Fig 3).

We obtained KEGG pathway annotation for 97,833 unigenes. A total of 60,906 sequences were assigned to 128 pathways. “Metabolic pathways (ko01100)” represented the largest group (13,741; 22.56%), followed by “biosynthesis of secondary metabolites (ko01110)” (7,136; 11.72%), “plant-pathogen interaction (ko04626)” (3,933; 6.46%), “plant hormone signal transduction (ko04075)” (2,745; 4.51%), “spliceosome (ko03040)” (2,275; 3.74%), and “RNA transport (ko03013)” (2,270; 3.73%; S1 Table).

Additionally, in the prediction of protein coding regions, the number of CDSs that were mapped to the protein database was 92,001, and the number of predicted CDSs was 7,012. The total number of CDSs was 99,013 (S3 Fig).

### Determination and functional categorization of differentially expressed genes (DEGs)

**Fig 4:**
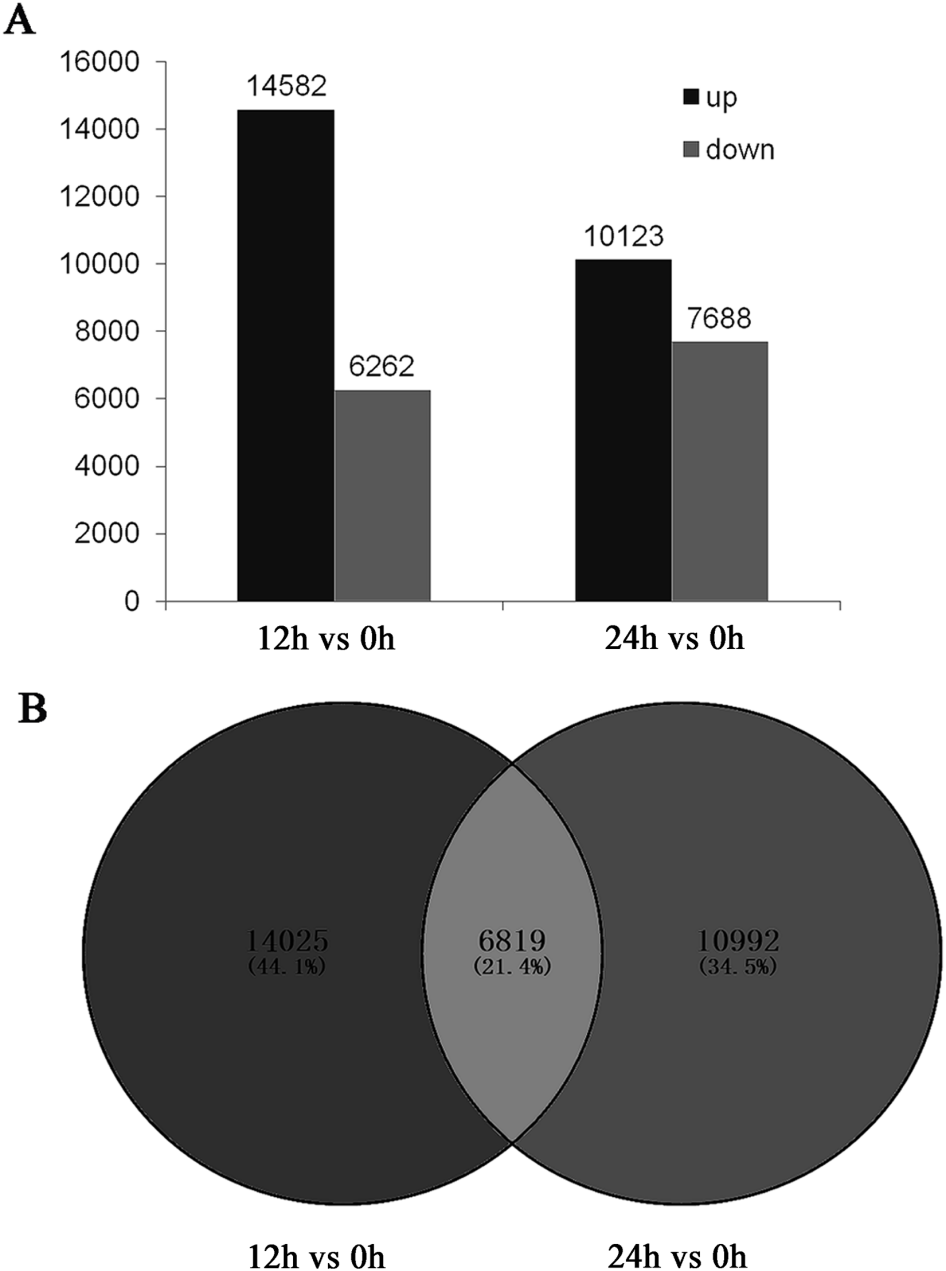
Statistical chart (A) and Venn diagram (B) of the DEGs of the transcriptomes in response to salt stress. The numbers of DEGs (both up- and downregulated) for “12 h versus 0 h” and “24 h versus 0 h” are listed.

To determine the genes showing altered expressions under salinity stress conditions, DEGs were defined as those with an FDR ≤ 0.001, a log_2_ ratio ≥ 1, and an RPKM > 2 in the compared libraries. We compared the 0 h library with the other two libraries (12 h versus 0 h and 24 h versus 0 h). As shown in Fig 4A, compared with the 0 h library, at the 12 h time point after salt treatment, the number (20,844) of DEGs was much larger than the number (17,811) at the 24 h time point. In the Venn diagram (Fig 4B) of those DEGs, it can be seen that among the genes that were differentially expressed at 12 h, only 48% were still differentially expressed at 24 h. In the figure showing the distribution of gene expression levels, the map corresponding to “12 h versus 0 h” exhibits a wider range than the map for “24 h versus 0 h” (S4 Fig). Therefore, there were many genes that responded to salinity stress in a very early stage, whose levels gradually returned to normal conditions.

All of the DEGs were categorized into 55 functional groups. The GO classification maps of the two comparisons were similar to each other. In these maps, among biological processes, the dominant groups were “cellular process”, “metabolic process”, “single-organism process”, and “response to stimulus”. In the cellular component category, the dominant groups were “cell”, “cell part”, “organelle”, and “membrane”. In the molecular function category, the dominant groups were “catalytic activity” and “binding” (S5 Fig).

The number of KEGG pathways corresponding to “12 h versus 0 h” and “24 h versus 0 h” was 126 in both cases, and the numbers of unigenes that were assigned to KEGG pathways were 9,899, and 7,769, respectively. After multiple testing corrections, we chose pathways with Q values ≤ 0.05 as significantly enriched among the DEGs. Then, we selected the useful significantly enriched pathways according to this criterion, resulting in 67 and 33 pathways, respectively (S2 and S3 Tables). The enriched pathways exhibiting more genes than the other pathways were “metabolic pathways (ko01100)”, “biosynthesis of secondary metabolites (ko01110)”, “ribosome (ko03010)” “Plant hormone signal transduction (ko04075)”, “plant-pathogen interaction (ko04626)”, “endocytosis (ko04144)”, “phenylpropanoid biosynthesis (ko00940)”, “glycerophospholipid metabolism (ko00564)” “glycolysis/gluconeogenesis (ko00010)”, “ether lipid metabolism (ko00565)”, “starch and sucrose metabolism (ko00500)”, and “oxidative phosphorylation (ko00190)”. These pathways were used as a reference when defining the useful genes under conditions of salinity stress.

### Detection of genes related to salinity tolerance

The identified DEGs are more likely to be related to salinity tolerance, and thousands of genes are induced or differentially expressed after salt treatment. Using the above tools in combination with previous reports, we selected some genes associated with salinity tolerance as relevant and divided them into categories. All of these genes are categorically listed in S4 Table. The main categories of the selected genes were as follows: transcription factors such as *bHLH* (basic helix-loop-helix), *DREB1A* (dehydration-responsive element binding protein 1A), *AP2/ERF* (APETALA2/ethylene response factor), *NAC* (NAM/ATAF/CUC), *AREB* (ABA responsive elements binding proteins), and *MYB2* (myb proto-oncogene protein); signal transduction molecules such as *CaM* (calmodulin), *YWHAs* (14-3-3 proteins) and *MAPKs* (mitogen-activated protein kinases); energy metabolism-related genes such as *AAC* (ADP, ATP carrier protein), *ALDH* (aldehyde dehydrogenase), *GTP* (Phosphoenolpyruvate carboxykinase), *GAPDH* (Glyceraldehyde-3-phosphate dehydrogenase), and *ADHs* (zinc-dependent alcohol dehydrogenase); various types of transporters, such as *SPs* (sugar transporters), *AATs* (amino acid transporters), *NRTs* (high-affinity nitrate transporters), and *MPTs* (mitochondrial phosphate transporters); proteins involved in Na^+^ exclusion and compartmentalization, such as *HKTs* (high-affinity potassium transporters), *SOS1* (salt overly sensitive), *NHXs* (Na^+^/H^+^ antiporters), and *AVPs* (H^+^ pyrophosphatases); aquaporins such as plasma membrane intrinsic proteins (*PIPs*), tonoplast intrinsic proteins (*TIPs*), and NOD26-like intrinsic proteins (*NIPs*); key enzymes involved in osmolyte synthesis, such as *TPS* (trehalose-6-phosphate synthase), *TPP* (trehalose-6-phosphate phosphatase), *mt1D* (mannitol-1-phosphate dehydrogenases), *MIP* (L-myo-Inositol-1-phosphate synthase), *BADH* (Betaine aldehyde dehydrogenase), *CHDH* (Choline dehydrogenase), and *P5CS* (delta-1-pyrroline-5-carboxylate synthetase); proteins related to water transportability and retention, such as *AQPs* (aquaporins), *LEAs* (late embryogenesis-abundant proteins) and *DHNs* (dehydrin); enzymes that cleave ROS such as *SOD* (superoxide dismutases), *MDHAR* (monodehydroascorbate reductases); cell wall proteins such as *HRGPs* (hydroxyproline-rich glycoproteins), *XETs* (xyloglucan endotransglycosylases), *AGPs* (arabinogalactan proteins), *PRPs* (proline-rich proteins) and *GRPs* (glycine-rich proteins); and photosynthesis-related proteins, such as *AdSS2* (adenylosuccinate synthetase 2), *KARI* (ketol-acid reductoisomerase), *NQO* (quinone oxidoreductase), *RPS4e* (small subunit ribosomal protein S4e), *LHCA4* (chlorophyll a/b binding protein 4), *psbA* (photosystem II protein D1), *psbO* (photosystem II oxygen-evolving enhancer protein 1), *psaD* (photosystem I subunit II), *psaH* (photosystem I subunit VI), *petA* (apocytochrome f), and *FAD* (ferredoxin-NADP^+^ reductase) (S4 Table). These genes will be discussed later.

### Verification of differential gene expression through qRT-PCR

**Fig 5:**
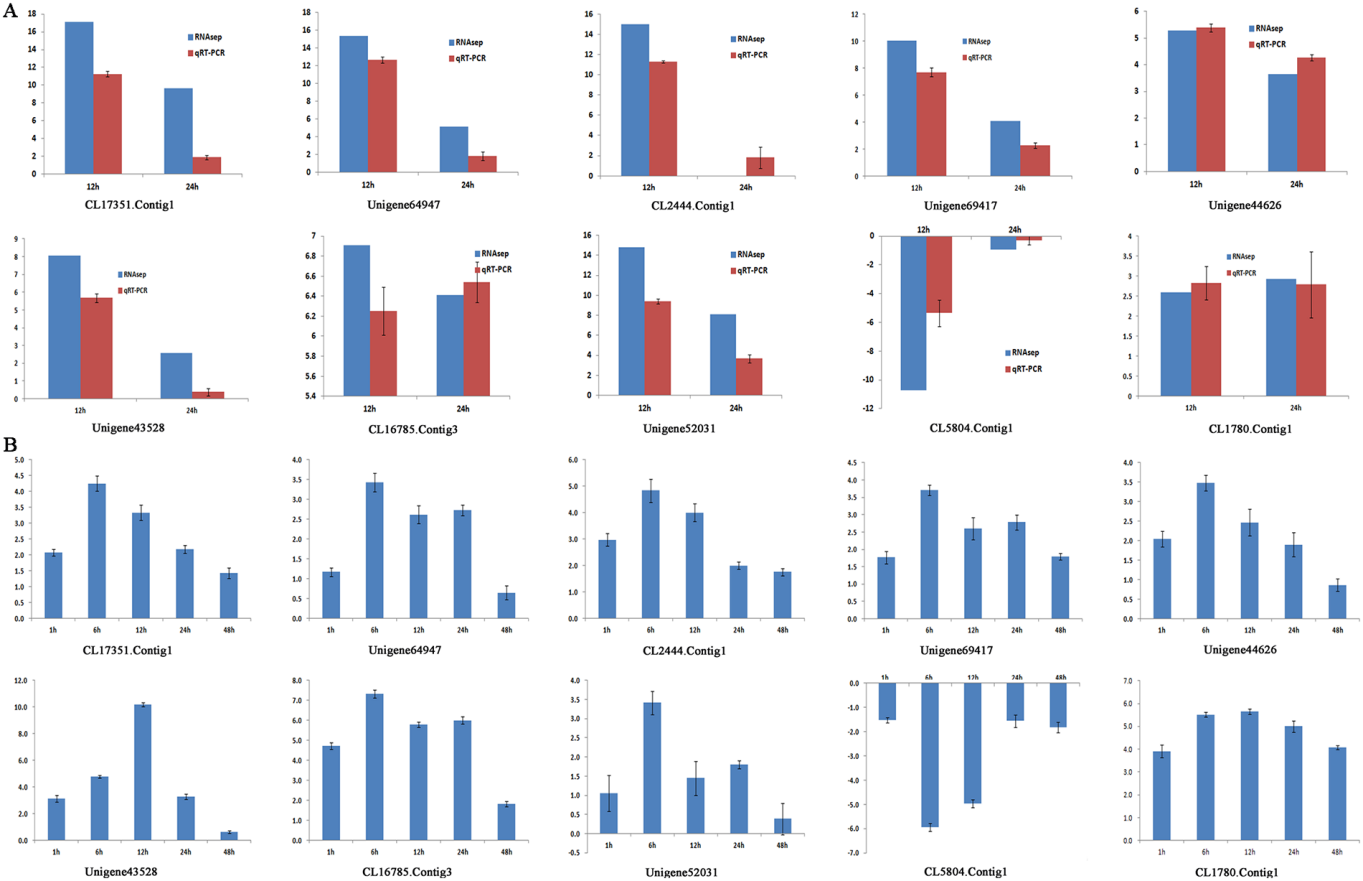
qRT-PCR analyses of unigene responses to salinity stress. The transcript abundance and expression patterns of selected genes were measured by qRT-PCR and RNA-seq at 12 h and 24 h and were compared with those at 0 h (Fig 5A). The transcript abundances of the selected genes were measured by qRT-PCR at 1 h, 6 h, 12 h, 24 h and 48 h and compared with those at 0 h (Fig 5B). RNA-seq values are the log2 values of the RPKMs of two libraries; the qRT-PCR values were determined via qPCR using the –ΔΔCT values.

To validate the results obtained from the Illumina sequencing data, 46 relevant unigenes were selected for quantitative real-time PCR (qRT-PCR) analysis with samples that were treated with 120 mM NaCl for 0 h, 12 h, and 24 h. The expression trends of the unigenes from the qRT-PCR and RNA sequencing analyses were consistent (S6 Fig). Ten of these Unigenes are shown in the text (Fig 5a), and their transcript abundances at 0 h, 1 h, 6 h, 12 h, 24 h and 48 h were also measured by qRT-PCR, demonstrating that these unigenes respond strongly to salt (Fig 5b). These results demonstrated that the transcriptomic profiling data accurately reflected the responses of *C*. *crassum* to salt stress.

## Discussion

### Global patterns of transcription in response to salt treatment

To improve the salinity tolerance of plants, researchers worldwide have performed myriad studies to elucidate how plants respond to salinity stress. However, salinity tolerance mechanisms are too complex for researchers to have achieved a full understanding as yet. Despite being limited by technologies and knowledge, studies on elite salinity-tolerant germplasms are helpful for improving the salinity tolerance of other germplasms. For example, over-expression of the *C*. *crassum* plasma membrane Na^+^/H^+^ antiporter gene *CcSOS1* improved the salinity tolerance of chrysanthemum ‘Jinba’ [12]. Therefore, functional genes from *C*. *crassum* could be used to improve the salinity tolerance of other species of chrysanthemums.

There have been many studies on the salinity tolerance of model plants, such as *Arabidopsis* and *Nicotiana tabacum* [26], and on some valuable crops with known genomes, such as *Oryza sativa* and *Zea mays* [27]. These studies have provided instructional theories and established components related to plant salinity tolerance, such as transcription factors, plant hormones, photosynthesis, osmolyte accumulation, ion elimination and toxicity alleviation. In cases where whole-genome sequencing data are lacking, NGS technology provides a powerful tool for transcriptome analysis of plants, and the *de novo* assembly of transcript sequences offers a rapid approach for determining expressed gene catalogs of non-model plants [28].

In this study, we performed sequencing analysis of *C*. *crassum* in response to salinity stress using the Illumina platform and *de novo* transcriptome assembly. Many DEGs were identified after salt treatment (Fig 4A). To better understand the function of the DEGs in leaves of *C*. *crassum* under salinity stress, the DEGs were categorized into 52 functional groups based on GO classification terms (S5 Fig). The ten most-enriched, dominant GO terms were “cell”, “cell part”, “metabolic process”, “cellular process”, “catalytic activity”, “organelle”, “binding”, “single-organism process”, “response to stimulus”, and “membrane”. These categories are also dominant in the transcriptomes of *Halogeton glomeratus* [29] and *Reaumuria soongorica* [30]. This result shows a similarity to other salt-resistant plants and emphasizes the remarkable salinity tolerance of *C*. *crassum*.

The DEGs determined in the two comparisons were also mapped to 128 pathways with KEGG annotations. By analyzing the obtained maps, we could not only identify which metabolic functions responded to salinity stress but also determined the particular genes in those metabolic pathways that were up- or downregulated. This provided us with conventional references for determining the functional genes and metabolic pathways involved in responses to salinity stress. For example, key enzymes involved in energy metabolism pathways (such as “gluconeogenesis (ko00010)”, “citrate cycle (ko00020)”, and “oxidative phosphorylation (ko00190)”); amino acid synthesis pathways (such as “alanine, aspartate and glutamate metabolism (ko00250)”, and “valine, leucine and isoleucine biosynthesis (ko00290)”); and DNA-, RNA- and protein-related pathways (such as “DNA replication (ko03030)”, “RNA transport (ko03013)”, “ribosome (ko03013)” and “protein export (ko03030)”) were upregulated; the genes encoding these enzymes were regarded as functional genes that respond to salinity. Using these tools, we selected functional genes and drew conclusions about strategies employed by *C*. *crassum* under salinity stress.

### Earliest-induced genes under salinity stress, such as transcription factors and signal molecules

Genes encoding transcription factors are some of the first genes to be induced under stress conditions [8]. Transcription factors are considered to have the greatest effect on crop salinity tolerance because these key transcription factors may regulate the induction or repression of a range of salinity tolerance genes. In the transcriptomes obtained in the present study, many reported transcription factors responding to salinity stress were found, such as *bHLHs* (Unigene40838, Unigene37206, and Unigene40839), *DREB1A* (CL6489.Contig1 and CL6489.Contig2), *AP2/ERF* (Unigene38328 and CL12828.Contig2), *NAC* (CL4758.Contig1, CL14612.Contig1, and Unigene60684), *AREB* (Unigene41165), *WRKY* (Unigene40047 and CL15510.Contig1), *GTL* (CL10054.Contig1) and *MYB2* (CL1046.Contig3 and CL1046.Contig1). All of these genes were upregulated at 12 h and returned to a low level at 24 h, and these genes have been reported to respond to stresses and regulate the growth conditions of plants under stress [31–33].

Additionally, some signal transduction molecules were found to be upregulated, such as *CaMs* (Unigene69398, Unigene70126, and Unigene70474), *YWHAs* (Unigene60260 and Unigene8553), and *MAPKs* (CL7742.Contig3, Unigene71806, and Unigene46385). These molecules have been reported to play a regulatory role in signal transduction [34]. These earliest-induced genes under salinity stress trigger a series of reactions related to stress endurance.

### Genes related to plant hormone transduction pathways were differentially altered under salinity stress

In the obtained transcriptomes, genes involved in hormone transduction pathways were differentially expressed. The four key enzymes in the abscisic acid transduction pathway (*PYL* [Unigene29811], *PP2C* [Unigene69780], *SnRK2* [CL1333.Contig3], and *ABF* [Unigene53263]) [7] were upregulated, as were key enzymes in the jasmonic acid transduction pathway (*COI1* [Unigene21774], *JAZ* [CL18161.Contig17], and *MYC2* [CL12169.Contig2]) [35] and key enzymes in the salicylic acid transduction pathway (*TGA* [Unigene21774], and *PR-1* [Unigene33280]) [36]. These three well-studied hormones respond to stresses, can trigger many reactions, and may react with growth-related hormones, resulting in morphological changes in plants [37].

Some of the identified genes in hormone signal transduction pathways were considered to present functions regulating the morphological changes observed in *C*. *crassum*. For example, key enzymes in the auxin transduction pathway (*TIR1* [Unigene46121], *AUX* [CL1780.Contig1], *ARF* [Unigene53204], and *SAUR* [CL16292.Contig2]) [38] were upregulated. Auxin plays an important role in coping with salinity and could help to maintain an ideal growth rate [39]. In the cytokinin transduction pathway [40], *A-ARR* (CL9399.Contig1) was downregulated, which could affect cell division [41], and leaf cells did not divide under salinity stress (Fig 1B). In the brassinosteroid transduction pathway [42], *TCH4* (Unigene38906, Unigene13917, and Unigene54097) was upregulated, which could contribute to cell elongation [43], while *CYCD3* (CL15069.Contig1, and Unigene61981), a cyclin that may contribute to cell division [44], was downregulated. These findings suggested that these three hormones played an important role in leaf incrassation and succulence in *C*. *crassum*.

### Genes that contribute to overcoming osmotic stress caused by salinity in *C*. *crassum*

Osmotic stress must accompany salinity stress [8]. In the transcriptomes of *C*. *crassum*, many water retention-related genes were identified. For example, aquaporins can regulate water transport and may have an important function (i.e., the detection of osmotic and turgor pressure gradients) [45]. There are many types of aquaporins in the plasma membrane and vacuolar membrane of plants, such as *PIPs*, *TIPs*, and *NIPs*. These aquaporins present distinct cell type- and tissue-specific expression patterns, and some of them respond to stress. However, their expression profiles vary under salinity stress, and some posttranscriptional mechanisms regulate aquaporin trafficking to the plasma membrane [46]. In our data, *PIP*s (Unigene72479, CL11177.Contig14, and CL392.Contig3) and *NIPs* (Unigene43528, CL6774.Contig4, and Unigene54541) were upregulated after salt treatment, while *TIPs* (CL1377.Contig1 and CL6342.Contig4) were downregulated after salt treatment. This means that different aquaporins showed different expression profiles under salinity stress in *C*. *crassum* and contribute to the regulation of water transport.

In addition, *LEAs* (Unigene52031, CL16208.Contig1, Unigene69354, Unigene70057, Unigene70137, and Unigene69772) and *DHNs* (CL3765.Contig11, Unigene44626, and Unigene33157) were significantly upregulated and were widely expressed after salt treatment. These *LEAs* and *DHNs* have been reported to be abundant proteins in plants that are highly hydrophilic and provide protection to plants under osmotic stress [47].

To improve osmotic pressure, some osmolytes must be synthesized to endure osmotic stress [8]. In our transcriptomes, a number of osmolyte genes were upregulated after salt treatment, such as *TPS* (Unigene42563, and Unigene14695), *TPP* (CL14450.Contig3, and Unigene45055), *mt1D* (CL11217.Contig1), *MIP* (Unigene55075, Unigene69121, and CL1330.Contig1), *BADH* (Unigene71886), *CHDH* (Unigene70102, Unigene69590, Unigene70406, and Unigene71308), and *P5CS* (Unigene44661, and Unigene41949). Additional enzymes participating in the synthesis of amino acids (such as *GPT* [Unigene70282], *GFPT* [Unigene4584] and *ABAT* [Unigene70080]) [48] and monosaccharides (such as *HK* [Unigene71535], *ALDO* [Unigene69763] and *GMDS* [CL9344.Contig1]) [49] were also upregulated. Amino acids and monosaccharide are types of osmolytes [50]. We also found that some enzymes that participate in the synthesis of ascorbate (including *GME* [CL9645.Contig2], *VTC4* [Unigene72075], and D-threo-aldose 1-dehydrogenase [Unigene71897]) [51] were upregulated after salt treatment. Ascorbate is a soluble carbohydrate that is abundant in leaves, and its accumulation would protect photosynthesis and improve the osmotic pressure of cells. Moreover, ascorbate is a cofactor for a large number of key enzymes and can influence both cell wall synthesis and hormone transduction [52]. Through the expression of the above genes, the plants were prevented from lacking water, and the ability of water to be taken up and retained was improved.

### Genes that contribute to the exclusion and compartmentalization of Na^+^

To prevent ion toxicity caused by salt, Na^+^ must be kept from accumulating to excessive levels in protoplasts. The results in Fig 2 show that there must be some metabolic pathways that control the exclusion of Na^+^ and the passive assimilation of K^+^. In our transcriptomes, F-type H^+^-transporting ATPases (Unigene65029, Unigene69416, Unigene69674, CL11916.Contig1 and Unigene69648) and V-type H^+^-transporting ATPases (Unigene69565, Unigene70189, Unigene70388, Unigene70530, and Unigene71306) were significantly upregulated after salt treatment. These H^+^-transporting ATPases establish an electrochemical H^+^ gradient across membranes to energize the membranes and participate in the synthesis of ATP [53, 54], thereby providing energy and electric potential for Na^+^ exclusion. Some ion transporters that have been frequently reported were identified in our transcriptomes, such as *HKTs* (CL15304.Contig2, and CL15304.Contig1), *SOS1* (Unigene38015), *NHXs* (Unigene12739, CL15766.Contig3, and Unigene12656), and *AVPs* (Unigene61687, Unigene61689, and Unigene61692). They were upregulated after salt treatment and shown to contribute to the reduction of the Na^+^ concentration in the cytoplasm [6].

### Reactive oxygen species (ROS) scavengers in the transcriptomes

Salinity also causes the production of ROS, which are noxious to plants [9], and *C*. *crassum* must control ROS to a concentration that does not do harm to plants [55]. *SOD* (Unigene40004, Unigene69671, Unigene70615, Unigene70870, Unigene64998, and Unigene70976), *MDHAR* Unigene69762, Unigene71436, and CL11284.Contig1), and *CAT* (Unigene70090, Unigene69612, Unigene69512, Unigene70251, and Unigene70939), which were significantly upregulated after salt treatment, provide effective protection to cells and ensure that many reactions in cells proceed normally.

### Genes contributing to the supply of energy and materials to the reactions triggered by salinity

Salinity causes many types of reactions in cells, and these reactions require energy and materials to function properly. Searching our transcriptomes revealed many energy metabolism-related genes, such as *AAC* (CL17351.Contig1, Unigene69746, and Unigene68723), *ALDH* (Unigene68789, Unigene69865, Unigene69866, and CL4198.Contig4), *GTP* (Unigene69854, Unigene56731, and Unigene68499), *GAPDH* (CL2444.Contig1, CL370.Contig1, CL2444.Contig2,and Unigene68703), and *ADHs* (CL14096.Contig2, Unigene71447, and Unigene69264), that were significantly upregulated after salt treatment and are helpful in supplying energy to other metabolic pathways to endure salinity stress. This is a common reaction of plants under stress [56, 57].

Some transporters, such as *SPs* (CL2828.Contig1, Unigene70725, and Unigene49470), *AATs* (Unigene69901, Unigene69792, Unigene69745 and CL14913.Contig1), *NRTs* (Unigene69733 and Unigene67760), and *MPTs* (Unigene64947, Unigene69759, and Unigene70273), were upregulated after salt treatment. These transporters supply materials to other metabolic pathways to endure salinity stress [58, 59].

The above genes were significantly upregulated at 12 h and returned to a low level at 24 h. Thus, mechanisms that scavenge genes that have performed their functions and have no need to remain active are expected to function at this stage. We found that some proteasome subunits (Unigene71130, Unigene70519, Unigene71062, Unigene70385, and Unigene71486) and some ubiquitin-conjugating enzymes (Unigene69417, Unigene30464, and CL15637.Contig1) were upregulated after salt treatment, which work as “cleaners” to digest unwanted mRNA and proteins [60] and return the expression of certain genes to normal levels (Fig 4 and S4 Fig).

### Behavior of the photosynthetic systems under salinity stress

The growth of plants must be supported by photosynthetic systems. Generally, in plants with less salinity tolerance, there is more damage to the photosynthetic systems under salinity stress [3]. After salt treatment, various parts of the photosynthetic systems of *C*. *crassum* showed different expression profiles. We found that components such as *RPS4e* (Unigene1713 and Unigene41867) and *LHCA4* (CL12376.Contig1 and CL13565.Contig1) of the “*LHC* (light-harvesting chlorophyll protein complex)”, which is responsible for trapping and transporting light energy to “photosystem I” and “photosystem II”[61], were downregulated, whereas components of “photosystem I” and “photosystem II” (such as *psbA* [CL6343.Contig11], *psbO* [CL8674.Contig2], *psaD* [CL5804.Contig1 and CL5804.Contig2], and *psaH* [Unigene58654]), which are responsible for oxidizing H_2_O to H^+^ and O_2_ [62], were downregulated. These changes may have been caused by the lack of water under osmotic stress. However, components of “cytochrome b6f complex”, “photosynthetic electron transport”, and “F-type ATPase” (such as *petA* [Unigene26782], *FAD* [CL14326.Contig2 and CL14326.Contig1], and F-type H^+^-transporting ATPase subunit gamma [Unigene43756]), which are responsible for mediating electron transport between PSII and PSI and converting the redox energy into part of the proton gradient used for ATP formation [62], were upregulated, as were key enzymes of the “carbon fixation in photosynthetic organisms” pathway (such as *FBA* [Unigene71038, CL13604.Contig2, Unigene69763, and Unigene35247], *PK* [Unigene71285 and Unigene62599], and *RPIA* [Unigene69514]) [63]. Thus, because of the difficulty in water uptake under salt shock, some components responsible for oxidizing H_2_O in the photosystem of *C*. *crassum* were downregulated, whereas other components were stimulated by the stress. At 24 h, all of these genes showed a tendency to recover to control levels. The previously mentioned ROS-scavenging enzymes provide protection to photosynthetic systems [55] and aid in the recovery of the photosynthetic systems of *C*. *crassum*. The recovery of the photosynthetic systems provides nutrition for the survival and growth of *C*. *crassum* under salinity stress.

### Explanation of the morphological changes in *C*. *crassum* based on transcriptome analysis

The growth condition under salinity stress is considered a main norm for judging the salinity tolerance of a plant, with a better growth condition indicating better salinity tolerance [39]. The growth rate of *C*. *crassum* (Fig 1A) after salt treatment showed no obvious decrease, indicating that this species exhibits remarkable salinity tolerance. Incrassation and succulence of salt-treated leaves that are often observed in halophytes such as *Salicornia europaea* [64] and *Populus euphratica* [10]. The leaves of C3 plants such as *Arabidopsis* have also been reported become slightly succulent after salt treatment [39]. As shown in Fig 1B, the leaves of *C*. *crassum* became obviously succulent after salt treatment, which mainly due to the expansion of each cell. It is common for plants to show some morphological changes under salinity stress, which will improve the adaptation of plants to stress. The morphological changes displayed in *C*. *crassum* are rarely observed in chrysanthemums, and it would be very novel and interesting to elucidate how these changes were initiated. Here, we discuss how the morphological changes observed in *C*. *crassum* are initiated based on transcriptome analysis. The genes contributing to morphological changes are listed in S5 Table.

As previously discussed, these morphological changes are mainly regulated by hormones such as auxins, cytokinins, and brassinosteroids (genes such as *TIR1*, *AUX*, *ARF*, *A-ARR*, *TCH4*, *CYCD3* and *SAUR* are hypothesized to be responsible). Additionally, the appropriate functioning of photosynthetic systems provides nutritional preconditions for the ideal growth of *C*. *crassum* (functional genes are *SOD*, *MDHAR*, *CAT*, *LHCs*, photosystem proteins, *FBA*, and *RPIA*). additionally, stress-induced hormones trigger certain components of metabolism, such as energy metabolism and transporters, to disrupt growth by depleting nutritional resources [65].

Plant cell expansion is often characterized as being the product of the opposing forces of intracellular turgor pressure (growth-promoting) and the turgor resistance of the cell wall (growth-inhibiting) [66]. As previously mentioned, we found that osmolytes accumulated to high levels and water absorbability and retention were improved in *C*. *crassum*. In this part, the mentioned genes are *TIPs*, *NIPs*, *PIPs*, *TPS*, *TPP*, *mt1D*, *MIP*, *BADH*, *CHDH* and *P5CS*. Therefore, as time went by, the “intracellular turgor pressure” became stronger.

We next consider the cell wall to examine “turgor-resisting forces”. The plant cell wall, and especially the cell wall proteins, undergoes adjustments under stress, as reported previously [67, 68]. The cell wall proteins exhibit functions involved in the morphogenesis and signal transduction of plants, and changes in the cell wall are a sign of cell differentiation [11]. In our transcriptomes, many types of cell wall proteins were found to be upregulated after salt treatment, such as *HRGPs* (Unigene69380, CL1283.Contig2, Unigene68945, and Unigene70662), which have an expansion-like domain and show a strong correlation with final cell length [69]. These expansions cause the cellulose microfibrils to slide apart and are considered to be the primary determinants of wall elongation [70], together with a group of enzymes known as *XETs* (CL10820.Contig2, CL10820.Contig3, and Unigene33390) [71], which were also upregulated after salt treatment. *PRPs* (Unigene69599, Unigene70083, Unigene70589, and Unigene70081) were also upregulated after salt treatment, which are extension proteins involved in the response to stress [72]. The adjustment of cell wall proteins of *C*. *crassum* under salinity stress showed a signal interaction and served as a marker for cell morphological changes. Furthermore, the expression of many extension proteins weakened “turgor-resisting forces”, making it easier for mesophyll cells to expand. Additionally, the cell wall proteins called *AGPs* (Unigene69390, Unigene68964, Unigene70408, and Unigene72883) were also upregulated after salt treatment; these proteins do not have structural function, but instead act as cell positional markers or as messengers in cell-cell interactions [73] Other upregulated genes included *GRPs* (Unigene68520, Unigene39763, and CL6740.Contig11), which play important roles in the development of vascular tissues and response to stresses [74].

With the expansion of cells, the membranes of the cells must also expand. In this context, the *LTPs* (Unigene70157, CL1283.Contig3, CL16785.Contig3, CL16785.Contig4, CL14640.Contig2, Unigene41619, and CL9481.Contig2) were also significantly upregulated and highly expressed after salt treatment, accounting for as much as 4% of the total soluble proteins of higher plants [75]. *LTPs* are thought to participate in membrane biogenesis, regulation of the intracellular fatty acid pools, cutin synthesis and responses to various stresses [76]. The abundance of this type of protein would benefit *C*. *crassum* in maintaining membrane stabilization and facilitating fatty acid-related pathways that would be helpful in stress tolerance.

### Genes responding to salinity without annotation

There were many genes with unknown functions without annotation in the databases that were significantly upregulated after salt treatment (such as Unigene43394, Unigene35778, Unigene69835, Unigene48169, Unigene69368, Unigene69480, Unigene51684, Unigene69811, Unigene48542, Unigene69615, Unigene69400, and Unigene68832) or were significantly downregulated (such as Unigene65383, Unigene65380, Unigene55783, Unigene65381, Unigene64461, CL12561.Contig1, CL16010.Contig4 and Unigene3931). The functions of these genes require further study.

## Conclusions

**Fig 6:**
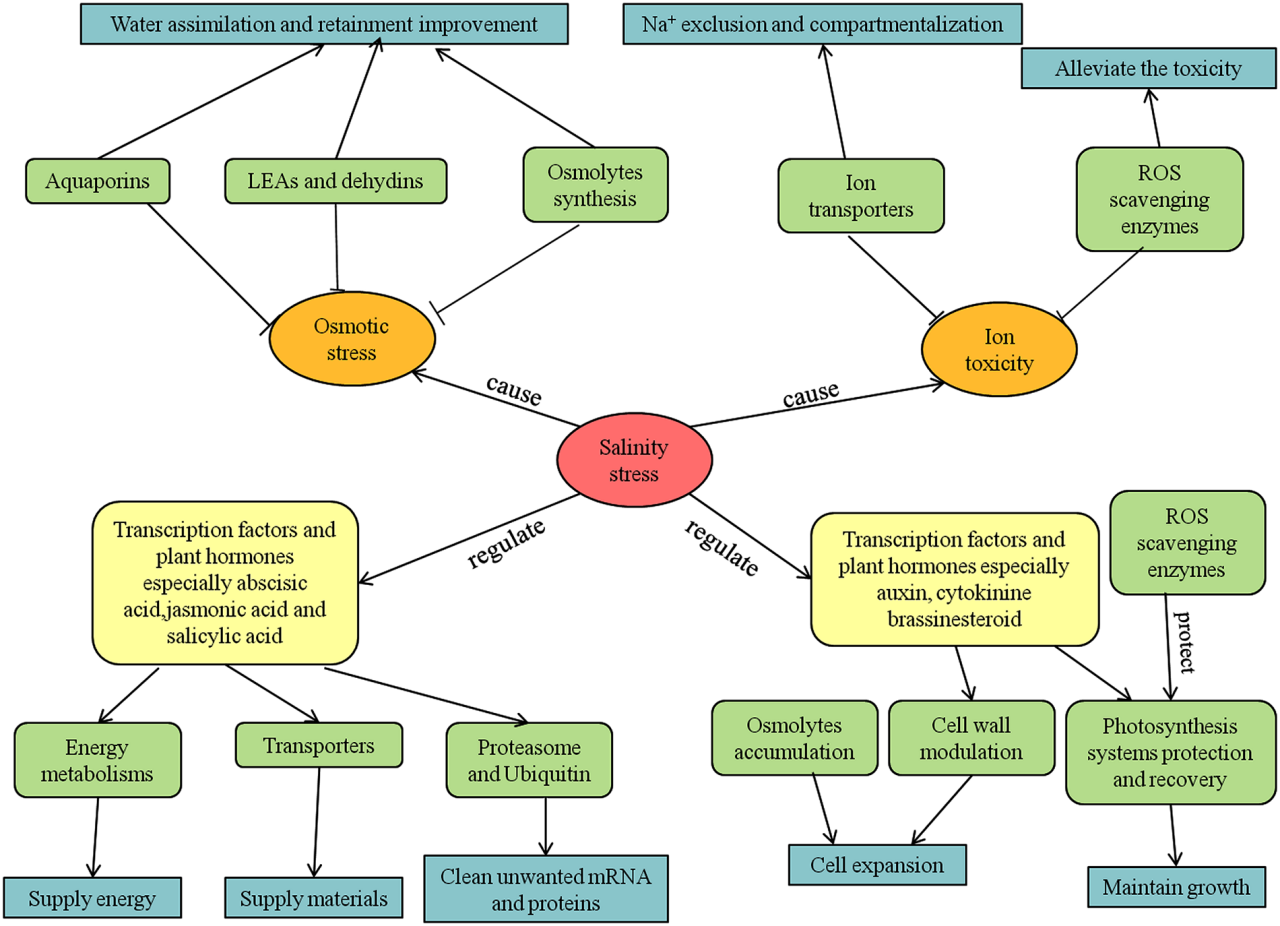
Mechanism of the *C*. *crassum* response to salinity stress.

Using the Illumina platform, we surveyed three transcriptomes of *C*. *crassum* leaves. We aimed to list the functional genes and pathways that contributed to salinity tolerance. Combined with the morphological changes observed under salinity stress, the results of the present study allowed us to infer and list the strategies adopted by *C*. *crassum* under salinity stress (Fig 6). Briefly, salinity stress first caused osmotic stress, which led to a lack of water. To maintain the water status of cells and cell functions effectively in *C*. *crassum*, large amounts of highly hydrophilic proteins, such as *LEAs* and *DHNs* were expressed, along with a reduction of water waste and regulation of the expression of aquaporins to sense turgor pressure and regulate water transportation. *C*. *crassum* also synthesized osmolytes to improve osmotic pressure. Osmotic stress was overcome by these strategies. Salinity also caused toxicity and perturbed the normal function of cell reactions. This toxicity mainly consisted of the accumulation of Na^+^ and the production of ROS. Accordingly, *C*. *crassum* expressed ion transporters to exclude and compartmentalize Na^+^ and expressed ROS-scavenging enzymes to remove ROS. The damage due to salinity was controlled by these strategies. The applied salinity stress triggered some transcription factors and plant hormones (especially some stress-induced hormones), including abscisic acid, jasmonic acid, and salicylic acid. *C*. *crassum* improved the activity of energy metabolism and transporters to supply energy and materials to other reactions to endure salinity stress. Additionally, *C*. *crassum* activated cleaners, such as proteasomes and ubiquitin, to remove unwanted mRNA and proteins. As time passed, the plants exhibited morphological changes to adapt to salinity stress, and these changes were initiated during an early stage of the stress treatment. The salinity treatment initiated changes in some transcription factors and plant hormones, including growth-related hormones such as auxin, cytokinins, and brassinosteroids. *C*. *crassum* expressed many types of cell wall proteins to expand the cell wall, and osmolyte accumulation and water storage increased turgor pressure. Therefore, the mesophyll cells of *C*. *crassum* expanded over time under salinity stress. To maintain normal growth, the photosynthesis systems of *C*. *crassum* were protected and recovered well under salinity stress. Through these strategies, *C*. *crassum* adapted to salinity and grew well.

## Supporting information

S1 Fig Growth conditions of *Chrysanthemum crassum* leaves under salinity stress. Salt: a, 0 d; b, 5 d; c, 10 d; d, 20 d; Control: e, 0 d; f, 5 d; g, 10 d; h, 20 d. Bar = 1 cm.
(TIF)

S2 Fig Characteristics of similarity searches of unigenes against Nr databases. E-value distribution of Nr annotation results (A). Similarity distribution of Nr annotation results (B). Species distribution of Nr annotation results (C).
(TIF)

S3 Fig All unigene CDS predictions. (DOC)

S4 Fig Distribution of differentially expressed genes in the two comparisons. The genes were classified into three categories. Red genes are upregulated, in that gene expression is higher in the right sample than the left sample. Green genes are downregulated, in that gene expression is higher in the left sample than the right sample. Blue genes are not differentially expressed. The horizontal coordinates are the expression levels of the right sample, and the vertical coordinates are the expression levels of the left sample.
(TIF)

S5 Fig Overview of GO function classification. (DOC)

S6 Fig qRT-PCR analyses of unigene responses to salinity stress. The expression patterns of selected genes were analyzed at 12 h and 24 h and compared with those at 0 h. RNA-seq values are the log2 values of the RPKMs of two libraries; the qRT-PCR values were determined via qPCR using the –ΔΔCT values.
(TIF)

S1 Table KEGG analysis of all unigenes. (XLS)

S2 Table KEGG analysis of DEGs for 0 h vs. 12 h. (XLS)

S3 Table KEGG analysis of DEGs for 0 h vs. 24 h. (XLS)

S4 Table Selected genes that responded to salinity stress. (XLS)

S5 Table Selected genes that related to the initiation of morphological changes. (XLS)
