# Supplementary figures and images for: Expression profiling of Chrysanthemum crassum under salinity stress and the initiation of morphological changes
Source: PLoS One. 2017 Apr 24;12(4):e0175972. doi: 10.1371/journal.pone.0175972 (PMC5402956; doi:10.1371/journal.pone.0175972)

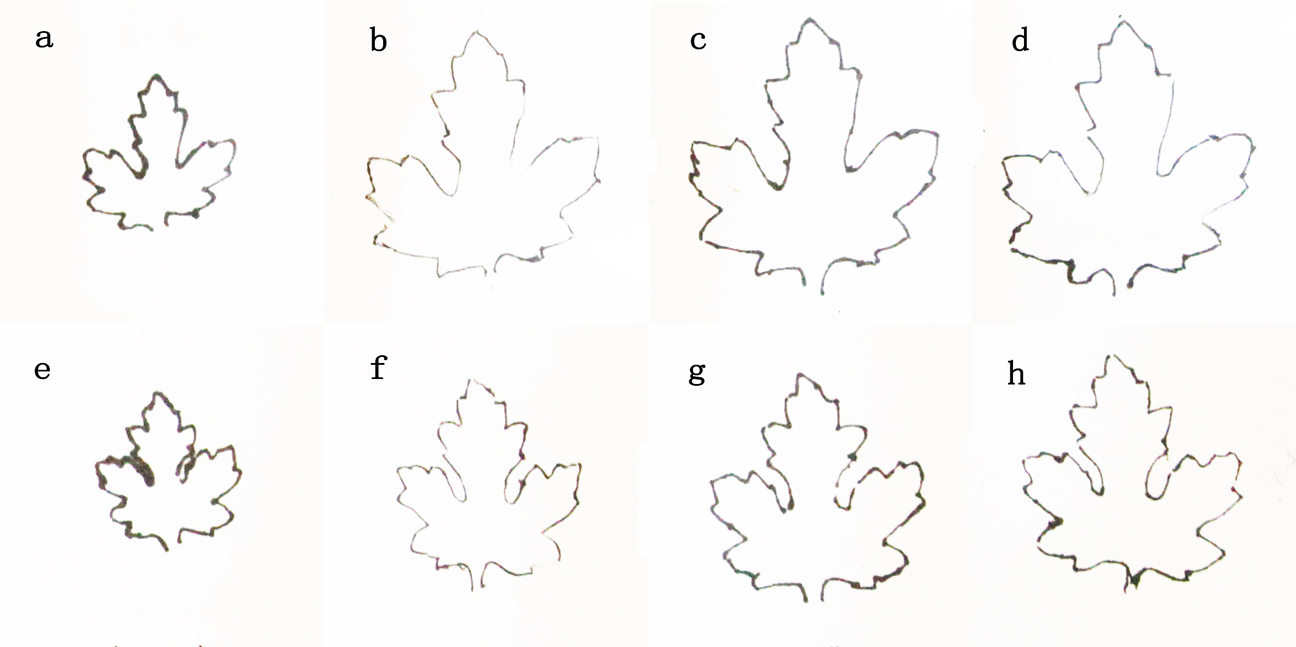

Supplement: S1 Fig — Salt: a, 0 d; b, 5 d; c, 10 d; d, 20 d; Control: e, 0 d; f, 5 d; g, 10 d; h, 20 d. Bar = 1 cm. (TIF) [file pone.0175972.s001.tif]

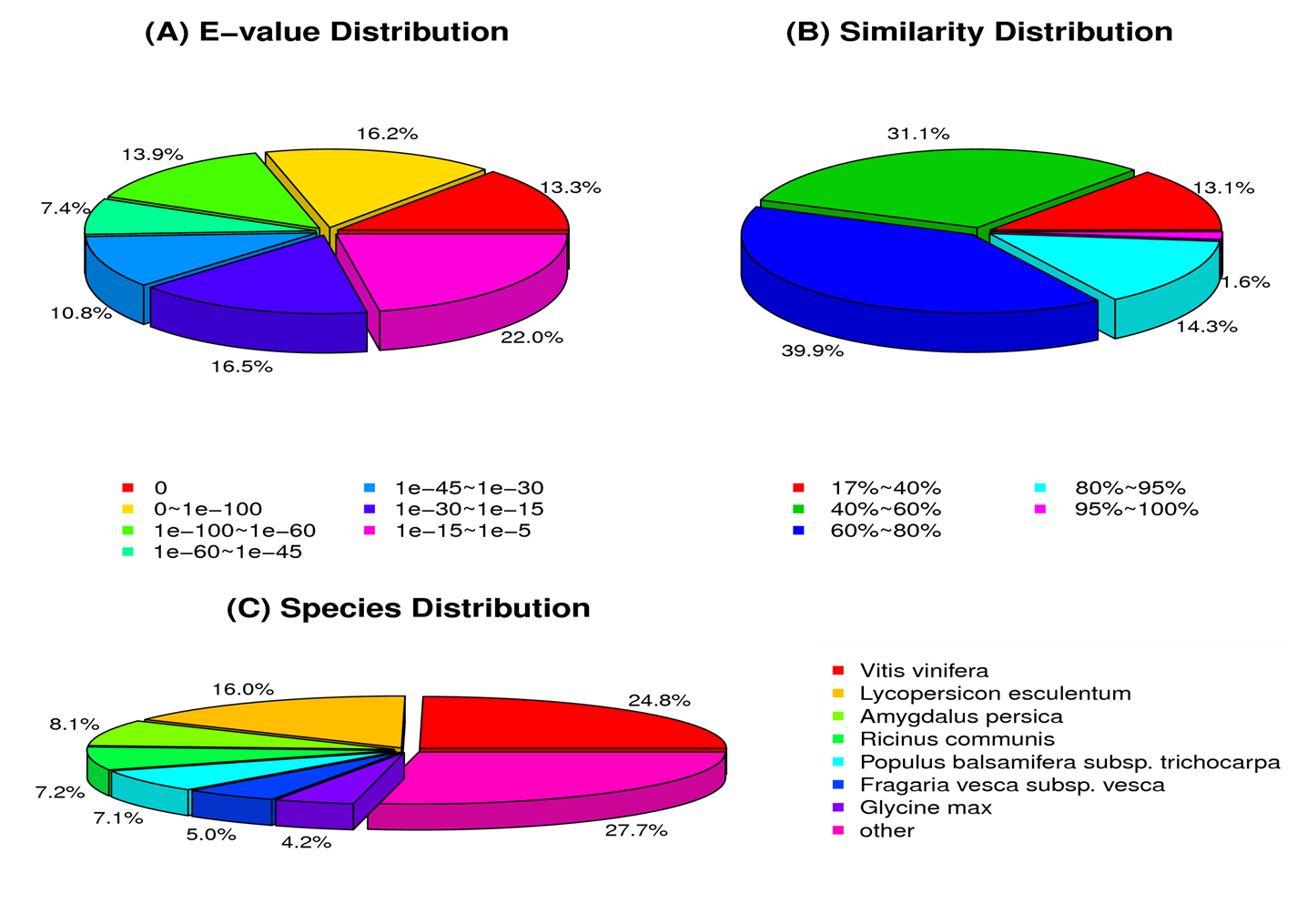

Supplement: S2 Fig — E-value distribution of Nr annotation results (A). Similarity distribution of Nr annotation results (B). Species distribution of Nr annotation results (C). (TIF) [file pone.0175972.s002.tif]

Additional file 2: Figure S1. All unigene CDS prediction.


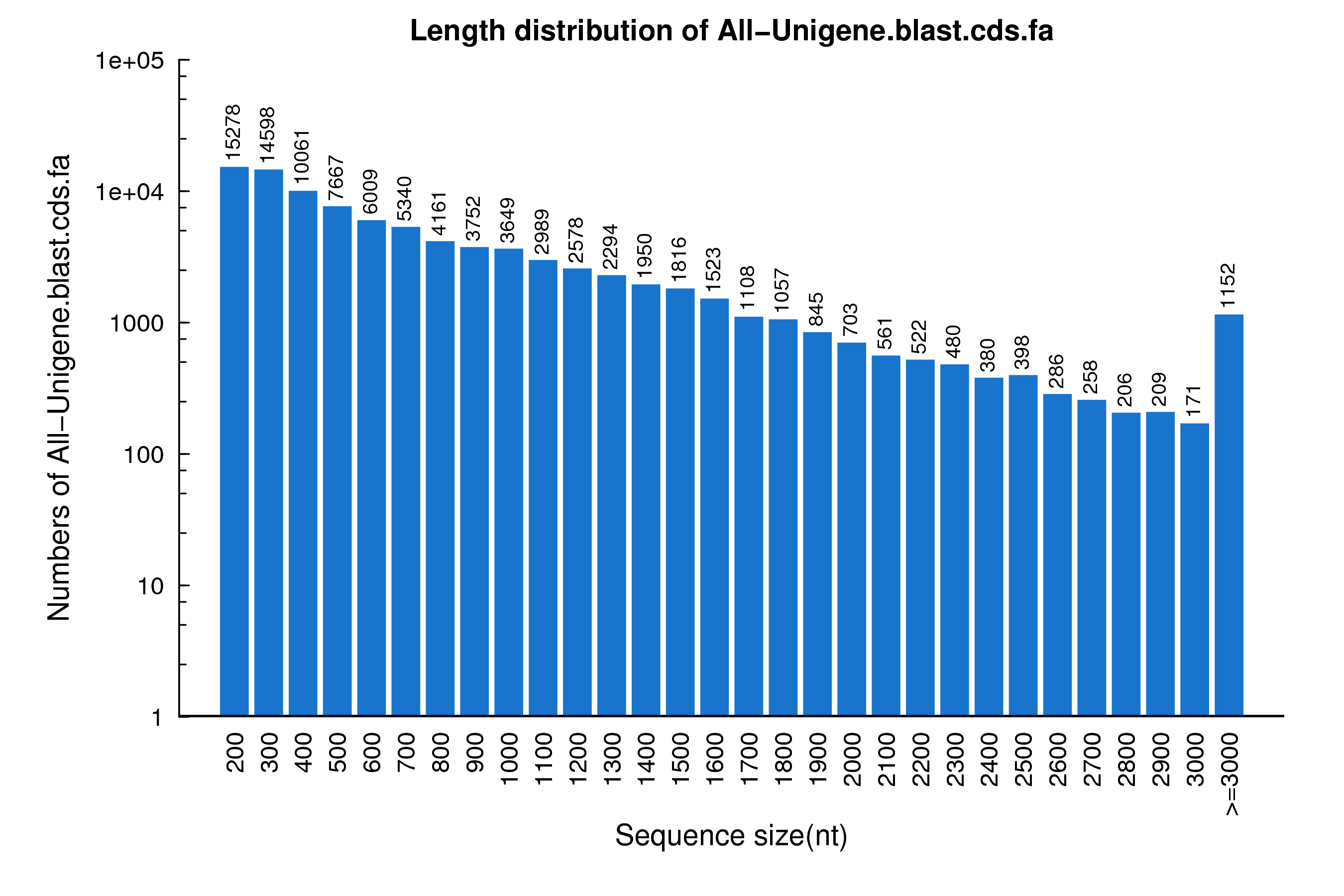


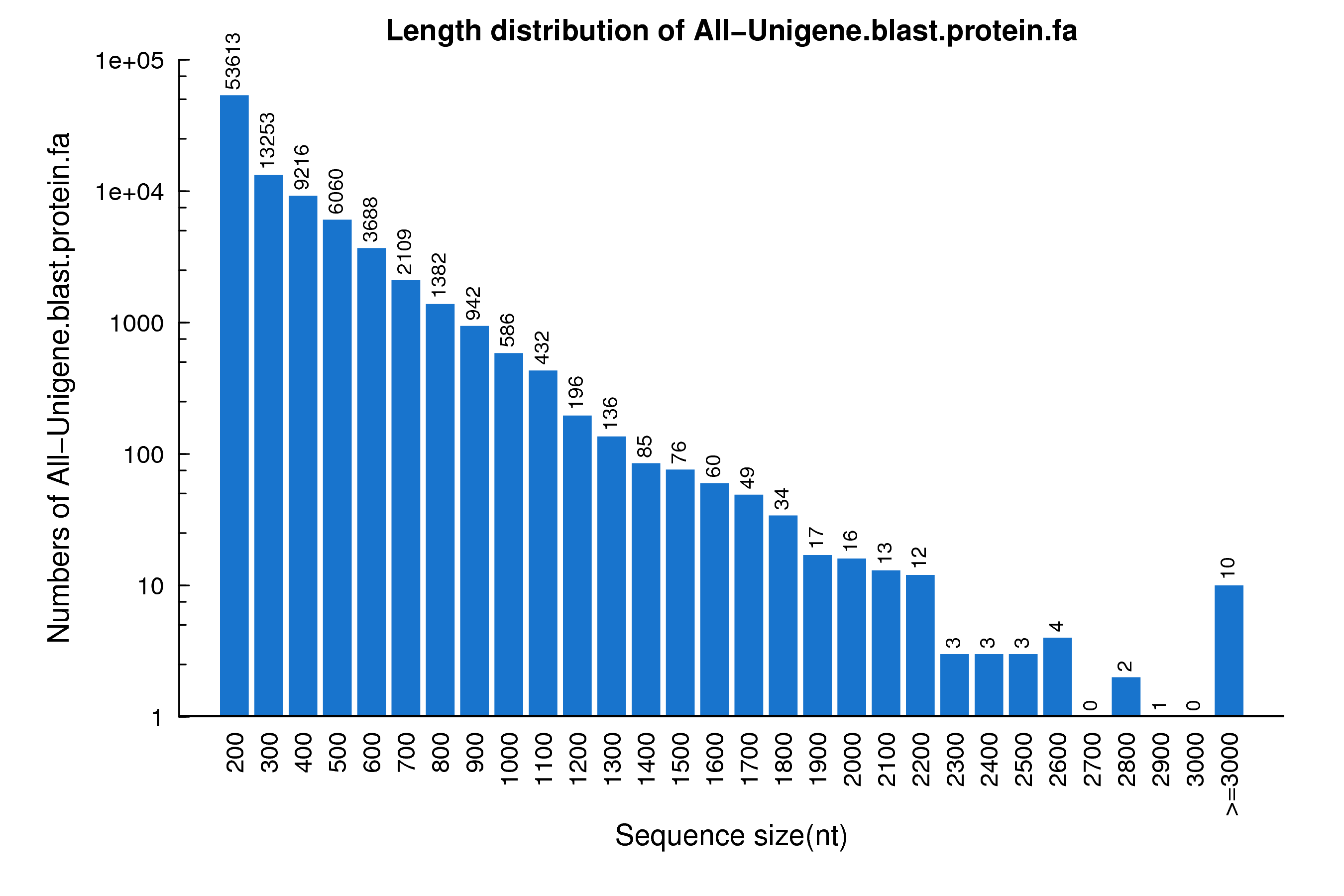


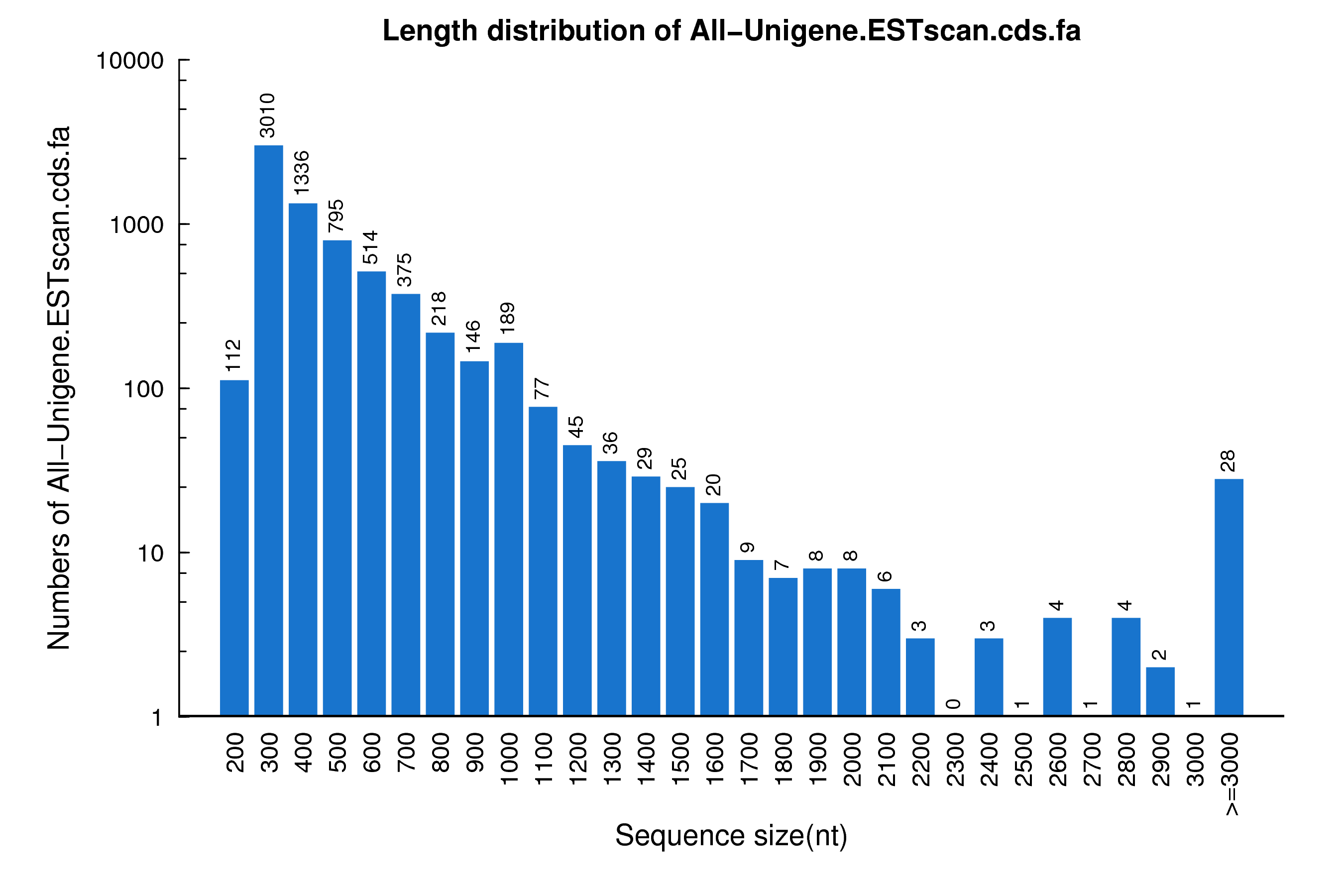


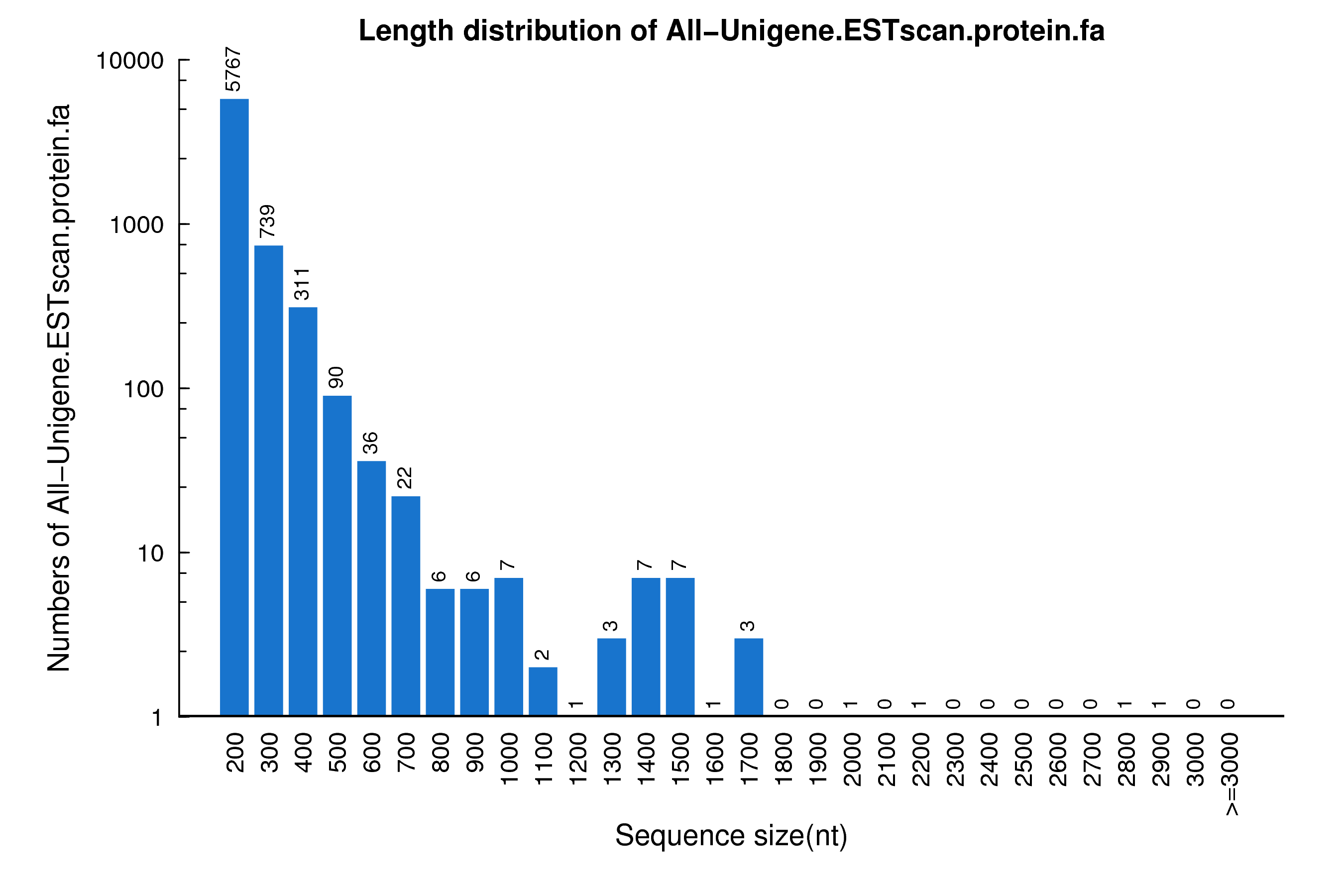

Supplement: S3 Fig — (DOC) [file pone.0175972.s003.doc]

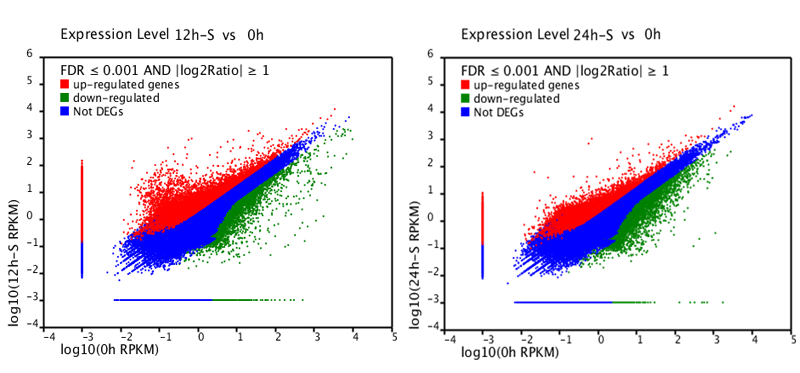

Supplement: S4 Fig — The genes were classified into three categories. Red genes are upregulated, in that gene expression is higher in the right sample than the left sample. Green genes are downregulated, in that gene expression is higher in the left sample than the right sample. Blue genes are not differentially expressed. The horizontal coordinates are the expression levels of the right sample, and the vertical coordinates are the expression levels of the left sample. (TIF) [file pone.0175972.s004.tif]

Additional file 2: Figure S1. Overview of GO function classification of DEGs.


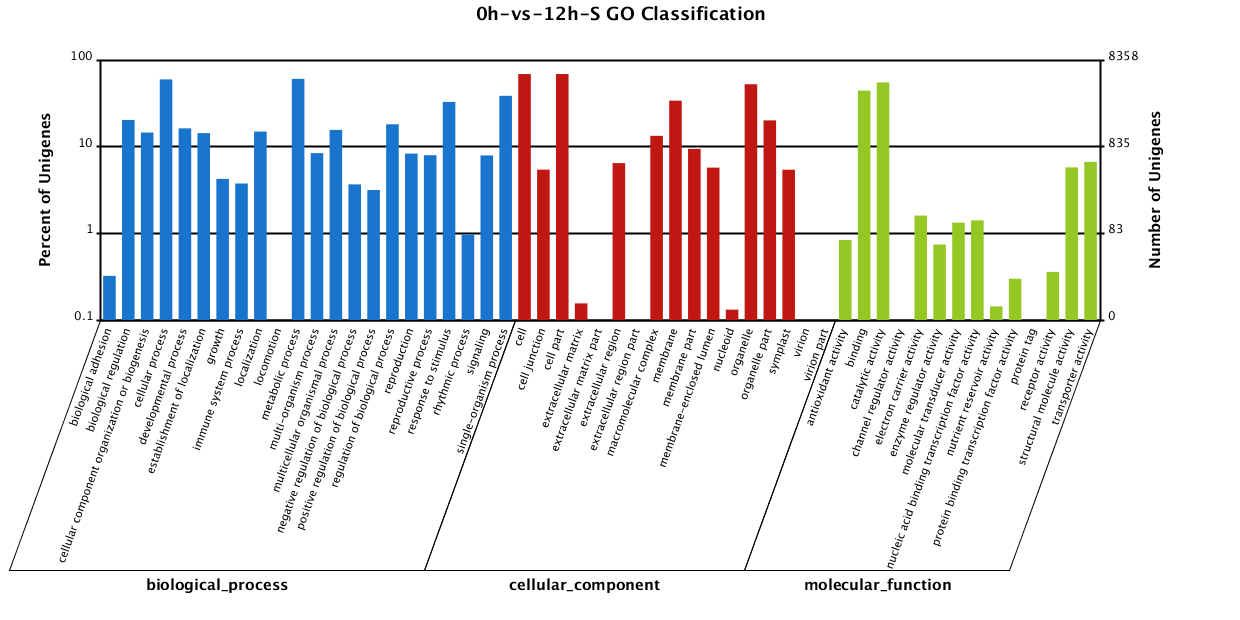


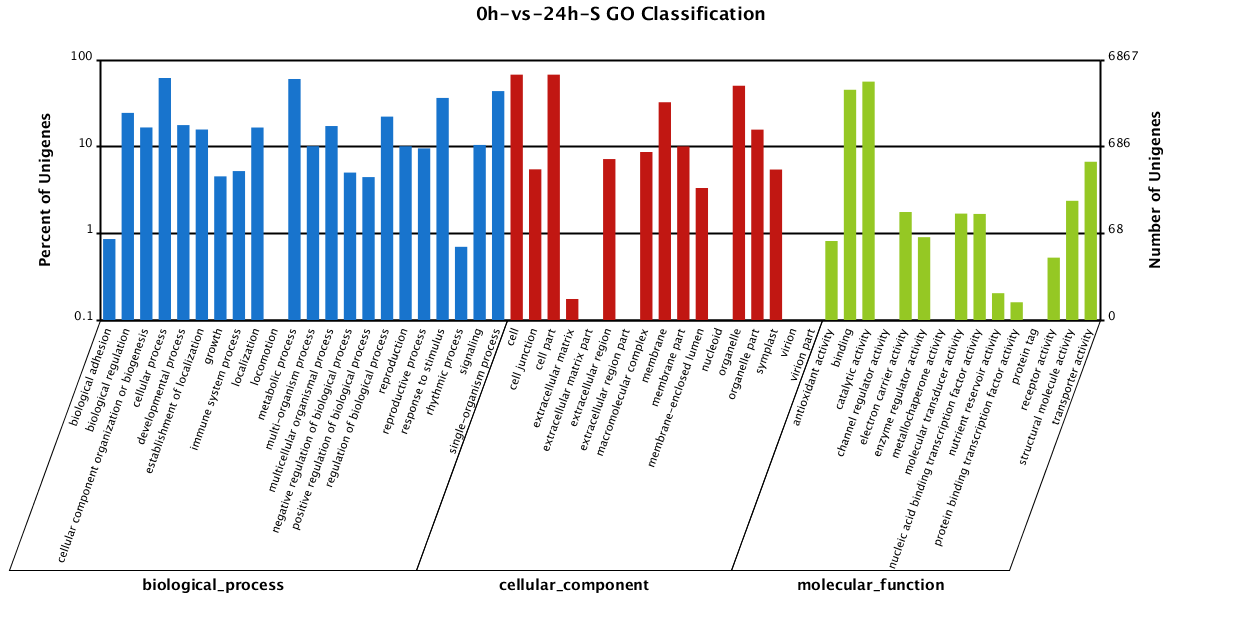

Supplement: S5 Fig — (DOC) [file pone.0175972.s005.doc]

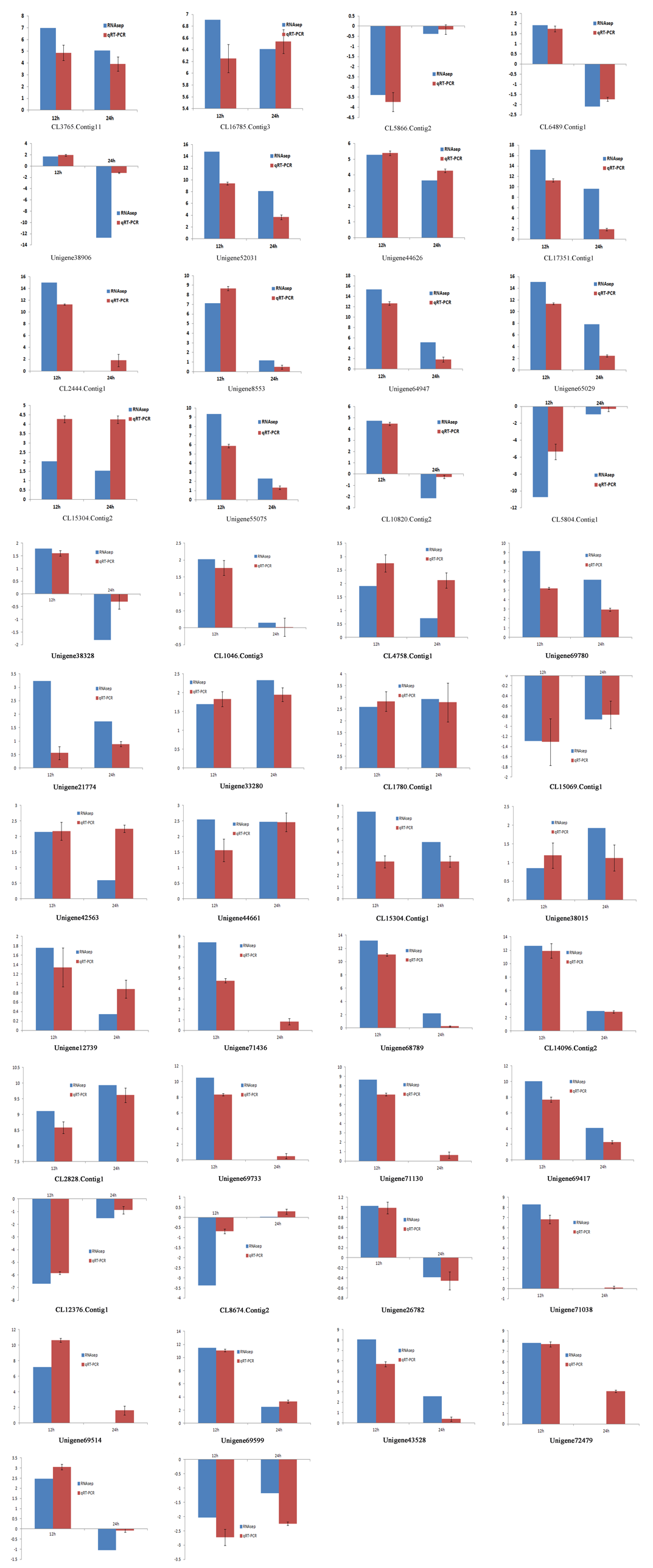

Supplement: S6 Fig — The expression patterns of selected genes were analyzed at 12 h and 24 h and compared with those at 0 h. RNA-seq values are the log2 values of the RPKMs of two libraries; the qRT-PCR values were determined via qPCR using the –ΔΔCT values. (TIF) [file pone.0175972.s006.tif]
